# Effectiveness of an Adult Food Literacy Program

**DOI:** 10.3390/nu11040797

**Published:** 2019-04-07

**Authors:** Andrea Begley, Ellen Paynter, Lucy M. Butcher, Satvinder S. Dhaliwal

**Affiliations:** 1 School of Public Health, Curtin University, Perth 6102, Australia; ellen.paynter@curtin.edu.au (E.P.); s.dhaliwal@curtin.edu.au (S.S.D.); 2 Foodbank Western Australia, Perth Airport 6105, Australia; lucy.butcher@foodbankwa.org.au

**Keywords:** food literacy, cooking, intervention

## Abstract

Nutrition education programs aim to improve food literacy domains covering the planning and management, selection, preparation and cooking and eating of healthy food. Reviews indicate programs are effective but acknowledge challenges with evaluation of community focused delivery. Food Sensations^®^ for Adults (FSA) is a free four-week nutrition and cooking program targeted at low-to-middle income Western Australians who would like to improve their food literacy. The aim of this research was assess how effective FSA is in changing food literacy and selected dietary behaviours. Statistical analysis identified a significant increase in postprogram scores for domains of planning and management, selection and preparation using factor scores (n = 1092). The proportion of the score increase in the postprogram scores compared to the preprogram scores was 10–25%. There was also a significant increase in self-reported fruit and vegetable serve intake, equating to an average increase of ¼ serve/day of fruit and ½ serve/day of vegetables. Of those classified as low food literacy, 61–74% improved postprogram scores in the three domains. FSA is effective in improving food literacy and dietary behaviours and the results add to the evidence base as to how effective these programs can be and for whom they should be targeted for future success.

## 1. Introduction

Nutrition education is the cornerstone of public health nutrition interventions [1], and increasingly there is a focus on improving practical food skills using experiential approaches. Food literacy has emerged as a term to conceptualise the knowledge, skills and behaviours required to achieve healthy dietary intake/diet quality covering four domains of planning and management, selection, preparation and cooking and eating [2]. Food literacy programs including cooking skill interventions are funded to address concerns about declining use of or devaluing of skills and therefore association with poor diet quality [3]. Programs work on the assumption that improvements in food literacy behaviours are likely to have positive impacts on dietary intakes as documented in logic models [4,5]. Reviews of programs addressing food literacy show a proliferation of community-based, government and other organisation funded efforts to teach people the planning, selection, preparation and eating behaviours thought to promote healthy diets [6,7,8,9]. Evaluation data from these programs need to be used in ways that can inform best practice in program delivery.

Systematic and other reviews of interventions focused on food literacy or those that have included a cooking component have found evidence of changes in confidence and behaviours and diet quality with a caveat that it is difficult to make conclusive comments on effectiveness due to a number of factors. Firstly, most programs are single group prospective designs as they are funded as community programs, not as research trials and do not include a control group [6,8,9,10]. Where a control group is included there is evidence that these programs show dietary intakes change significantly [9] where it is measured, as either self-reported dietary behaviours or total dietary intakes are not always included in the evaluation [7,8]. Secondly, there is wide variability in the duration, target groups, program curriculums and, generally, small sample sizes that limit the ability to make conclusions about what these programs do and how they work [9]. Most studies to date include some hands on cooking experiential learning component and this is usually in addition to other components such as nutrition education which may provide an indication of pragmatic approaches to design [9,11]. Programs report a wide range of sessions (3-year to multiyear), and evidence of application of theoretical basis for change and definitive logical models of relationships between inputs and outcomes is limited [4,12,13]. Finally, evaluation designs without validated tools are common.

The proliferation of program delivery is occurring at the same time that research is still elucidating the conceptualisation and measurement of food literacy [14,15]. There is currently no consensus on the best way to measure food literacy behaviours for program delivery and there are a number of efforts looking to validate monitoring and surveillance measurement tools [15,16,17]. The food literacy behaviours referred to as food resource management targeted by the US Expanded Food and Nutrition Education Program (EFNEP) have been used as the basis for evaluation for over thirty years and the 10–15 item food behaviour checklist is used extensively by other programs [18,19,20,21]. EFNEP is delivered typically over 8 to 12 weeks and is effective at increasing self-reported positive behaviours [22] with statistical analysis of pre–post evaluation demonstrating changes in mean scores. There is a lack of detail on how effective these programs are when segmenting participants on enrolment to focus on those with low food literacy at the start and who benefits most from the program.

The Foodbank of Western Australia (WA) has invested in food literacy programs since the mid-nineties in an effort to improve the nutritional status of disadvantaged populations [23]. Foodbank WA’s *Food Sensations^®^ for Adults* (FSA) is an adult food literacy program targeting individuals from low-to-middle income households who would like to increase their food literacy skills. The program is promoted as a free nutrition and cooking program with participants recruited through existing community groups or able to self-enrol in public programs. Programs are delivered primarily face to face and videoconferencing is used to extend the reach into regional areas. FSA is marketed extensively to all adult Western Australians using social and traditional media, websites, health professional referral and word of mouth. The only inclusion criterion is the ability to shop and cook independently.

FSA was first implemented in 2011, but underwent extensive redevelopment in 2015 to align with an Australia Food Literacy Model [2] and Best Practice Criteria for Food Literacy Programs commissioned by the Western Australian Department of Health (Department) [24]. The current version of FSA, funded by the Department was contracted for a period of two and half years up until June 2018. The contract service level outcomes include increased food literacy knowledge, skills and confidence and increased intentions to regularly select, prepare and eat nutritious foods. Programmatic funds supported independent external evaluation which has enabled further analysis of service level data to contribute to the evidence base for food literacy program effectiveness. A reference group of stakeholders with members from the Department, including the WA Country Health Service, community organisations and other informants review the evaluation data and provide advice on directions for program delivery twice a year.

**Figure 1:**
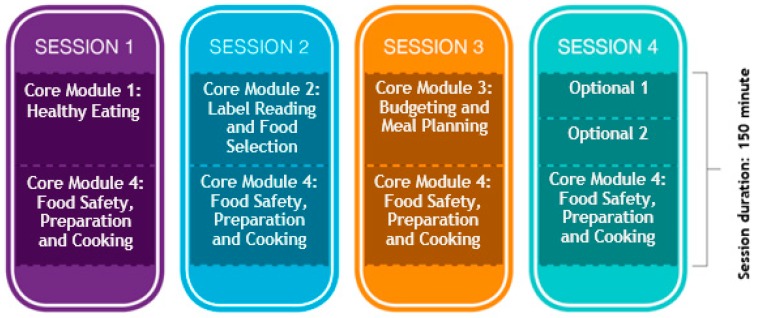
Food Sensations for Adults (FSA) Program Curriculum.

FSA is a four session program, each session with a two and half hours in duration equating to ten hours of contact time for each program (see Figure 1).

The program’s curriculum comprises of eight lesson plans that are divided into four core modules and four optional modules. All curriculum lesson content has been mapped to the four domains of food literacy (Planning & Management, Selection, Preparation & Cooking and Eating) and 11 components of food literacy outlined in the empirically tested Australian Food Literacy Model [2]. The four core modules taught over the first three sessions were developed to address all 11 components of food literacy. These sessions cover the Australian Guide to Healthy Eating food groups, using the nutrition information panel and other features of food labels to select healthier foods and budgeting tips, and suggested ways to plan meals and each week participants prepare, cook and eat several recipes demonstrating healthy eating and budget friendly meals and snacks. Optional modules are offered in session four, to reinforce the food literacy components and enable to the contextualisation of content to meet the needs of various different subgroups of participants [25]. The groups may select one of the four optional modules to be delivered as part of the program. The optional modules are as follows; Healthy lunchboxes and snacks, Healthy mind, healthy body, Supermarket tour and Gardening for health. These optional modules relate directly to the four domains of food literacy being covered by the program. Hands-on cooking, offered in the second half of every session, is over half the program duration allowing participants to learn and practice basic cooking skills in safe environment and taste new foods, while preparing healthy recipes. Foodbank WA have produced a number of visual cookbooks, designed specifically for low literacy groups, and each participant receives at least one recipe book to take home, with the intention of encouraging continued healthy home cooking. Sessions are tailored for each group’s abilities in consideration of recipes to be cooked, and facilitators have a strong commitment to delivering relevant, informative and practical information.

The delivery of the FSA program is guided by the Health Belief Model (HBM) as well as Social Learning Theory. These models have been utilised to ensure the program moves beyond the simple dissemination of information to include strategies to build confidence, self-efficacy and motivation. The program emphasises the link between poor diet and risk of chronic diseases, and therefore the benefits of a healthy diet using the constructs of the HBM perceived susceptibility, perceived severity, perceived benefits and perceived barriers to influence behaviour change [26]. Perceived barriers are addressed through the provision of reassurance and strategies to overcome diet and nutrition related obstacles. The nutrition education component of the program operates as a cue to action and the individual goal setting activity encourages self-efficacy as a key factor for behaviour change [27]. Both observational learning, practice and repetition of skills and knowledge will also contribute to behaviour change which in turn builds self-confidence [27]. Participants who attend FSA observe the facilitators and their peers cooking and enjoying nutritious foods. These observations drive the individuals to model this positive behaviour at home [28] and within their wider personal environment. Additionally, adult learning considerations have been included to increase the likelihood of participants attempting to modify dietary intake outside of the session’s context. The program’s design aims to maximise educational impact by providing multiple opportunities for the application of critical thinking skills, experiential learning and demonstration of technical proficiency to create a sense of accomplishment.

The purpose of this article is to assess how effective FSA is in changing food literacy and selected dietary behaviours. The paper objectives are to (1) establish the relationship between pre and post food literacy behaviour scores, (2) assess if there a significant change between mean pre and post food literacy and selected dietary behaviour scores, (3) determine if the program is effective at moving participants most at risk of low food literacy and finally (4) identify variables associated with different food literacy components in participants who move from low food literacy behaviour.

## 2. Materials and Methods

### 2.1. Study Design

Participants attending 223 FSA programs run between May 2016 and June 2018 were encouraged to complete pre and postprogram questionnaires prior to starting the first session and on completion of the last session (n = 2628). The majority of participants were from existing community groups in the metropolitan area (60.3%), followed by metropolitan public programs (15.3%), and nearly one-quarter were from regional programs (24.3%). Not all programs were able to be evaluated for various reasons, including limitations relating to mental health, disability and low English language proficiency, in addition some participants did not provide consent. There was no reimbursement for completing questionnaires.

### 2.2. Evaluation Tool

The items for the pre and post-program questionnaires were developed to address the funder’s required service level outcomes and included a 14 item behaviour checklist referred to as a food literacy behaviour checklist and four short closed-ended questions on dietary behaviours to measure change. The preprogram questionnaire included additional items including four food literacy-related practices, a question on reasons for enrolment and eight socio-demographic variables. The development and validation process for the preprogram food literacy behaviours questionnaire has been published elsewhere [29], including the considerations of respondent burden, cognitive load and reading levels of potential participants. The food literacy behaviour questions were developed from adapting an extensively tested food behaviour checklist used in EFNEP [18,19,20,30]. Three food literacy related practice questions included in the preprogram questionnaire were selected from the Department’s Nutrition Monitoring Surveillance Survey (NMSS) [31], covering level of household responsibility for choosing and preparing meals and shopping similar to those used in the US National Health and Nutrition Examination Survey [32] and self-rated cooking skills drawn from unpublished qualitative research to inform the Go for 2&5^®^ fruit and vegetable social marketing campaign [33]. An additional food literacy-related practice question was included on attitude to cost of healthy foods to measure one objective required by the funder. Four short dietary questions were adapted from the same survey series, including two questions on average consumption of serves of fruits and vegetables and two questions on the frequency of consumption of fast food meals and sugar-sweetened drinks. Demographic characteristics collected from participants included sex, age, highest education level, household composition, postcode, birth in Australia and identifying as Aboriginal and/or Torres Strait Islander. Income as a primary demographic characteristic was extrapolated from self-reported postcode and converted to the Australian Bureau of Statistic’s Socio-Economic Indexes for Areas (SEIFA) decile ranking of the Index of Relative Socio-economic Disadvantage [34]. Deciles 1 to 4 were considered low-income, 5–7 middle-income and 8–10 high-income. A final question on the reason(s) for attending was included in order to identify participant intentions.

### 2.3. Food Literacy Behaviour Scores

Exploratory factor analysis of 13 questions in the questionnaire relating to food literacy behaviour was carried out previously and successfully identified three food literacy behaviour factors: Plan & Manage, Selection and Preparation [29]. The factor loading cut-off used was 0.4, which meant that 11 questions were included in one or more of the three factors. Factor scores for each of the three food literacy behaviour factors were calculated for each individual both pre- and postprogram. Possible responses to each question, Never, Sometimes, Most of the time and Always were scored one to four, respectively. This response score was multiplied by the factor loading for each question, and the factor score was calculated by summing the values of each included question [35].

The first step was to create groups of participants using the preprogram food literacy behaviour scores for the three factors or domains. Quantiles are used to create cut-off values within a distribution of variables [36]. This method has been used with population or patient groups in epidemiological and clinical studies to determine those more at risk of specific outcomes. When a specific cut off value is unknown, a defined variable is used to specify tertiles, where the population is split into three groups [37]. Variables such as biomedical scores, clinical tests or dietary intake are used to define the lowest tertile group, the group with the highest risk or poor outcomes associated with the low score. In this study the lowest tertile of food literacy behaviour factor scoring participants, were defined to be the low food literacy behaviour group for each factor, those most at risk of low food literacy [38]. Tertile cut-offs for low, moderate and high scores were defined using the preprogram scores for each of the three factors and these same values were used for the postprogram factor cut-off scores to assess participant change across groups. The three factors have been kept separate in the analysis to demonstrate the performance of each and not to assume they are equally weighed in their contribution to overall food literacy.

### 2.4. Statistical Analysis

Data were analysed using SPSS (IBM) version 25. Results were considered statistically significant if *p* < 0.05. The second step was to explore the relationship between pre- and postprogram factor scores. Correlation of determination (R^2^) were calculated to determine the proportion of variation in postprogram scores that could be accounted for by the preprogram scores. To examine the effectiveness of the program in improving food literacy behaviour scores, paired *t*-tests were carried out using pre to postprogram scores. To investigate change in self-reported dietary intake, paired *t*-tests were used to compare intake pre and postprogram both in fruit and vegetable serves. To explore change in reported frequency of fast food meal and sugar-sweetened drink consumption pre- to postprogram, McNemar’s and McNemar–Bowker tests were utilised [39]. These tests were also utilised to examine the movement of participants between the low food literacy behaviour group and the moderate or high food literacy behaviour groups based on pre or postprogram scores.

FSA aims to ensure that people improve food literacy behaviours, so the focus for analysis was to assess the transition from the preprogram low tertile group to the moderate and high groups postprogram. Within each food literacy behaviour factor—Plan & Manage, Selection and Preparation—participants who stayed in the low scoring tertile postprogram were compared to those who moved from the low tertile into the moderate or high tertile. Multivariable logistic regression was undertaken to determine which variables together contribute to the shift from low food literacy behaviour scores preprogram to moderate or high food literacy behaviour scores postprogram. Variables included in the analysis were those collected in the pre and/or postprogram questionnaires and outlined above; eight on sociodemographic information, four on dietary intake, three on food literacy-related practices, one on the attitude towards the cost of healthy food and one on the reason for attending.

The majority of responses to these questions (15) were recorded as categorical values. Self-reported fruit and vegetable intake in serves were coded as continuous variables. For the questions relating to self-reported dietary intake, the preprogram scores were subtracted from the postprogram scores to produce a change in intake. The change in frequency of fast food meal and sugar-sweetened drink were recoded as either increased, no change or decreased from pre- to postprogram. Results are shown with an odds ratio: this indicates the likelihood of a person who has moved from low to moderate-high food literacy behaviour score choosing this answer compared with the reference answer (indicated by the 1). An odds ratio greater than one with a significant *p*-value shows an increased likelihood. As change in self-reported fruit and vegetable intake were continuous variables, an odds ratio greater than 1 indicates for every one serve increase, the likelihood of this individual being one who improved their food literacy behaviour score to moderate-high rather than remaining low.

### 2.5. Ethics

Ethics approval was obtained from the Human Research Ethics Committee at Curtin University (RDHS-52-16). Participants were provided with a verbal explanation of the purpose of the research at the start of their first session and a written research information sheet. Written consent was obtained prior to questionnaire administration.

## 3. Results

### 3.1. Response Rate and Demographic Characteristics

Questionnaire data was collected from 1850 participants—1625 preprogram (87.6%) and 1319 postprogram (71.1%)—resulting in 1092 participants providing both pre- and postprogram data (59%). The missing data in the questionnaires were random and no questions were commonly missed. Participants were more likely to be female (81.1%), not in paid employment (67.1%) and from low-to-middle income areas, as determined by postcode (72.4%) (see Table 1). There was a range of age groups and household compositions. Less than a quarter had completed a bachelor degree or higher (23.7%). Cultural diversity is evidenced as just over half had been born in Australia (57.8%) and 6.2% identified as Aboriginal or Torres Strait Islander.

### 3.2. Food Literacy Behaviour Change

There was a positive and linear relationship between pre- and postprogram scores for each of the three factors. Correlation of determination scores (R^2^) were 0.398, 0.165 and 0.280 for Plan & Manage, Selection and Preparation, respectively. These values indicate that the preprogram score accounts for 40%, 17% and 28% of variation in postprogram scores, for Plan & Manage, Selection, and Preparation factors respectively.

Paired *t*-tests comparing pre- and postprogram scores identified a statistically significant increase in postprogram scores for all three factor scores (*p* < 0.0001) (Table 2). The proportion of the score increase in the postprogram scores compared to the preprogram scores was 25.1% for Selection, 11.8% for Preparation and 9.7% for Plan & Manage. There was also a statistically significant increase (*p* < 0.0001) in self-reported fruit and vegetable serve intake, increasing by 15% and 24% respectively. This equated to an average increase of ¼ serve of fruit and ½ serve of vegetables.

Self-reported fast food meal and sugar-sweetened drink intake also was also significantly different pre and postprogram (*p* < 0.0001). Of those reporting three or more times a week fast food meal consumption preprogram, only 30% reported this postprogram, 70% reported a lower frequency (Table 3). Of those reporting once or twice a week preprogram, 50% reported the same intake postprogram, 6% a higher frequency and 44% a lower frequency. Participants that reported less than once per week preprogram, 53% reported the same intake postprogram, 19% reported higher intake and 29% reported a lower intake. Eighty percent of participants reporting never preprogram remained in this category postprogram and 20% reported a higher frequency.

Of the program participants initially reporting consuming sugar-sweetened drinks three or more times a week, 50% reported a decrease in intake and 50% reported the same intake postprogram (Table 4). Of those reporting once or twice a week sugar-sweetened drink intake preprogram, 48% reported the same intake postprogram, 41% reported a lower intake and 10% a higher intake. Similarly, 48% of participants who initially reported less than once a week sugar-sweetened drink intake reported the same intake postprogram, 36% reporting a lower intake and 16% a higher intake. Eighty six percent of individuals reporting never to consuming sugar-sweetened drinks preprogram also reported this postprogram and 14% reported a higher consumption.

For all three food literacy behaviour factors there was statistically significant difference in the proportion of low food literacy behaviour participants in the pre- and postprogram populations (Table 5) (*p* < 0.0001). Of the participants consider to be low scoring preprogram for plan & manage, 61% were in the moderate or high group postprogram. Conversely, less than 4% of participants that initially were moderate or high scoring became low scoring postprogram, and 96% of participants who were initially in the moderate or high scoring group remained there postprogram. Similarly, for selection, almost three quarters (74%) of low scoring participants preprogram shifted to moderate or high postprogram. Those who were moderate or high scoring preprogram, more than 92% remained in the moderate or high category postprogram. Less than 8% shifted from preprogram moderate or high groups to the low scoring group postprogram. Those participants initially low scoring for preparation, 65% shifted to the moderate or high groups postprogram. Over 93% of those who were moderate or high scoring at the start stayed in the moderate or high group postprogram. Less than 7% shifted from the moderate or high groups preprogram to low scoring group postprogram.

### 3.3. Associations with Food Literacy Behaviour Improvement

Multivariable regression analyses identified variables characterising participants who improved their food literacy score over the course of the program from the low scoring tertile to the moderate or high group. Two dietary intake variables were associated with an improvement in Plan & Manage food literacy behaviour scores (Table 6). Individuals improving their Plan & Manage score were unlikely to report drinking more sugar-sweetened drink at the end of the program; they were more than 2.5 times more likely to either decrease or not change their reported intake in sugar-sweetened drinks, rather than increase. They were likely to report a larger improvement in serves of vegetables, a participant reporting a one serve/day increase was 1.4 times more likely to also improve their food literacy score to the moderate or higher group by the end of the program.

Three sociodemographic variables were associated with an increase in selection scores: sex, household composition and being born in Australia (Table 4). Those participants who started the program with low Selection food literacy behaviour score but moved to moderate or high after the course of the program were 2.7 times more likely to identify as male and 2.1 times more likely to be born outside of Australia. They were 4.7 times more likely to live as part of a couple rather than living alone. Two answers relating to interest in attending the program were found to be associated with an increase in selection score; these individuals were 2.5 times more likely to select learn how to read food labels and 2 times more likely not to select make healthier snacks and lunchboxes for children.

Four variables were associated with an increase in preparation scores (Table 6). Individuals improving their preparation score initially from low to moderate or high postprogram were 2.7 times more likely to live in a high income area and 14.9 times more likely to report no responsibility for meal preparation. They were 8.5 times more likely to report decreased sugar-sweetened drinks intake postprogram compared with preprogram, rather than increased intake.

A number of variables did not statistically significantly contribute to the improvement any food literacy behaviour scores. These included four sociodemographic variables: age, education level, employment status and identifying as Aboriginal or Torres Strait Islander. Other variables that were not significant were responsibility for doing the household food shopping, self-reported cooking skills, attitude towards the cost of healthy foods, change in self-reported fruit intake and self-reported fast food meals and several reasons for attending.

## 4. Discussion

FSA is effective in improving self-reported mean change in food literacy behaviours in three domains and selected dietary behaviours. Further examination of the program impact on participants who are classified as low in food literacy behaviours on enrolment has confirmed the effectiveness of the program as the majority of these participants move to moderate or high food literacy groups. We have established different variables associated with participants who move from low food literacy in the three domains. By examining three domains of food literacy behaviours we were able to distinguish different variables that are associated with participants who move from preprogram low food literacy to postprogram moderate or high food literacy. Strengths of the evaluation are the large sample size and a response rate comparable and some instances greater than other similar community programs [40,41,42,43]. The evaluation has addressed a number of the acknowledged limitations addressed at the start of this paper. The majority of participants were enrolled through existing community groups which should reduce selection bias as most participants in programs are considered convenience samples as they select to participate, such as those interested in cooking [7,8] or potentially looking for food [44]. Additionally the results used questions as part of the measurement tool originating from other program research proving validity of using a behaviour checklist approach and specific validity was confirmed before further analysis [29,45]. Similarities in questions enables the results to be assessed against other programs which have used a behaviour checklist to compare impact, particularly those studies with a quasi-experimental design [22,46,47]. Our analysis has extended beyond the typical presentation of results as change in group means.

### 4.1. Food Literacy Behaviour Change

The effectiveness of food literacy programs is challenging to determine partly as the wide range of duration, curriculum, target groups and evaluation designs make comparisons difficult [9]. FSA is demonstrating comparable results as similar programs. For example, Cook Well was a seven week cooking skills intervention study for socioeconomically deprived adults in the UK [48]. It was one of the first programs to measure components of food literacy behaviours such as confidence with cooking to explain the impact of such programs. Cook Well showed a small but positive effect on confidence in cooking with basic ingredients and following a recipe. The Jamie Oliver’s Ministry of Food UK eight-week cooking program was effective in increasing cooking confidence for adults of lower socioeconomic status and producing positive qualitative results for food resource management [43]. Similarly an online nutrition education program with low-income women was found to increase use of a grocery budget when shopping for food, and increase confidence in managing money to make healthy foods available [49].

The FSA food literacy behaviour changes are confirmed when compared to programs with quasi-experimental design or nonequivalent comparisons group and evaluate the program with a similar food behaviour checklist. This gives confidence that the changes evidenced in FSA are valid. EFNEP’s Eating Smart Being Active, a minimum nine-week curriculum showed a mean positive behaviour change score for food resource management, food safety and nutrition [22]. Cooking Matters, a six-week nationally delivered US program demonstrated positive impact on food resource management when compared to a nonequivalent comparison group [46]. Healthy Choices for Every Body a minimum 7-week curriculum used the food behaviour checklist to prove that changes in food resource management were statistically significantly higher for the intervention group [47].

### 4.2. Dietary Behaviour Change

FSA’s pre- to postprogram evaluation demonstrated significant change in serves of fruit and vegetables and frequency of sugar-sweetened drinks and fast food meal consumption; although it should be noted participants reported low serves and low frequency of consumption at program enrolment. The change in dietary behaviours reported here is similar to other programs and suggests a maximum change that can be expected. A systematic review assessing healthy eating interventions found that in low income groups there are small intervention effects when compared with controls to a change of just under half a serve of fruit and vegetables but slightly larger effects in general populations [50]. Fruit and vegetable intake is the most commonly assessed food group impact from programs with a cooking component and mostly positive changes are reported [7,9]. Sugar-sweetened drink consumption is less likely to be measured and does not typically show a change pre- to postevaluation and limited evaluation of frequency of fast or takeaway food consumption provides some evidence that this can change as a result of these types of programs. FSA’s increases in fruit and vegetable consumption has been found in other programs such as the Jamie’s Ministry of Food Australia and UK programs [41,43]. Jamie’s Ministry of Food Australia eight-week program, found a statistically significant increase 0.52 serve/day increase in vegetable consumption and 0.28 serve/day of fruit was shown postprogram where the control group did not change. That program was designed to reduce consumption of takeaway foods/meals and ready-made meals found a statically significant reduction in takeaway food consumption postprogram but no change in pre-prepared meal intake.

### 4.3. Participants with Maximum Improvement

Knowing who is benefiting the most from food literacy programs will assist with the marketing and enrolment processes in addition to providing evidence for target group decisions for funders. Effectiveness can be judged by the majority of low literacy participants on enrolment moving to moderate or high food literacy. This result contributes to understanding the different domains of food literacy and what parts of the program (plan and manage, selection and preparation) are important for different subgroups of participants. Improving planning and management behaviours appeared important for improving diet quality (higher vegetable intakes and lower sugar-sweetened drinks). Selection behaviour improvements were successful for those who indicated they had come to the program to learn to read food labels and for males, couples and those born overseas. A criticism or limitation of programs is the high enrolment of females and findings that show gender differences assist in tailoring programs to different groups [7]. Previous findings from FSA related to those with low selection scores being statistically more likely to be born outside Australia is potentially related to being unfamiliar with food labelling, types of foods and supermarkets [38]. FSA was successful in teaching people to read food labels to support healthy food selection. Preparation skill improvement was associated with those from high income-classified postcodes which is a reminder to not assume that low socioeconomic groups are more likely to have low food literacy. FSA appears to assist those with no current responsibility in their household for choosing and preparing meals to improve in the preparation domain. More needs to be known about the impact on participants who start with high food literacy as evident in their self-reporting of the frequency of planning, selection, preparation and eating behaviours. Programs may operate to reinforce or confirm for participants that they are doing the right thing [51].

FSA is an example of a program grounded in the available evidence base and a response to specific government policies and funding decisions to improve diet quality and health outcomes. Programs need to be contextualised to the populations they target. On one hand, the published program evidence criticises the generalisability of program results due to lack of uniformity in design, target groups, curriculum and duration [9]. At the same time, evidence of country differences in food literacy behaviours [52,53] and profiling or segmenting subgroups in the population results in different programs [38]. It may not be possible to produce recommendations on the ideal food literacy program from the evidence base but we need to consider what is best practice for different countries and contexts [24]. Research on the implementation fidelity of programs including quality will assist in informing this tension in program type and maximum effectiveness. Programs which provide opportunities to practice new behaviours and try new tastes based on behaviour change techniques are likely to be more effective [54]. Questions related to quality, dosage or amount, reach and participant responsiveness need to be asked to address this gap in the literature [55].

### 4.4. Limitations

There are several points to consider in the generalisability of these results. The study design consisted of a single group of participants enrolled in a food literacy program who consented to complete the evaluation. Self-selection bias needs to be considered in the types of participants who choose to enrol and/or complete evaluation. It is likely that participants who did not complete evaluation were from culturally and linguistically diverse groups. The number of questions assessing the domains of food literacy was limited in the questionnaire development and validation as respondent burden, cognitive load and reading level of the target group were considerations [29]. The questions were all closed-ended using scales of responses. There may be other variables associated with domains and low literacy that were not tested. Participants self-reported and this may also lead to a social desirability bias and/or produce a response-shift bias. A response-shift bias may occur because questions are asked at two different time points and perceptions or a different frame of reference results in differentiate question interpretation [45]. The findings do need external validation.

## 5. Conclusions

Developing and reinforcing food literacy confidence and behaviours that are central to supporting healthy food selection and preparation should lead to improvements in diet quality and ultimately health outcomes. We have taken an innovative approach to food literacy program evaluation not done previously to our knowledge. The results have gone further to segment the participants at enrolment to focus on those with low food literacy behaviours at the start of the program to support the conclusion that FSA is an effective food literacy program. These results add to the evidence base as to how effective these programs can be and for whom they should be targeted to for future success.

## Figures and Tables

**Table 1:** Demographic characteristics of participants.

Characteristic	Responses	FSA Respondents: Pre and Postprogram Questionnaire
**Sex**	Male	205 (18.9%)
(n = 1084)	Female	879 (81.1%)
**Age**	18–25 y	127 (11.7%)
(n = 1085)	26–35 y	247 (22.7%)
	36–45 y	263 (24.2%)
	46–55 y	150 (13.8%)
	56–65 y	140 (12.9%)
	66 and over	158 (14.6%)
**Household Composition**	Couple with children	388 (35.9%)
(n = 1081)	Single person	185 (17.1%)
	Partner	205 (19.0%)
	Single parent with child/children	105 (9.7%)
	Other: Family/Extended family/Shared/Supported accommodation	198 (18.3%)
**Education level**	Certificate/Diploma/Trade	374 (34.8%)
(n = 1076)	Finished high school	258 (24.0%)
	Bachelor or higher	255 (23.7%)
	Some secondary/finished primary	187 (17.4%)
**Employment status**	Unemployed/unable to work	263 (24.5%)
(n = 1073)	House duties/maternity leave/retired	457 (42.6%)
	Part-time/casual	245 (22.9%)
	Full-time/self-employed	107 (10.0%)
**Socioeconomic Index ^1^**	Low	468 (44.7%)
(n = 1048)	Middle	290 (27.7%)
	High	290 (27.7%)
**Born in Australia ^2^**	Yes	597 (57.8%)
(n = 1032)	No	435 (42.2%)
**Identify as Aboriginal or Torres Strait Islander ^2^**	Yes	63 (6.2%)
(n = 1022)	No	960 (93.8%)

^1^ SEIFA, Socio-Economic Indexes for Areas, derived from postcode [34]. ^2^ Added in later version of questionnaire.

**Table 2:** Paired *t*-tests comparing pre- and postprogram factor scores for the three food literacy behaviour factors and change in self-reported dietary intake of fruit and vegetables.

	Pre (Mean)	Post (Mean)	*p*-Value	95% CI Lower	95% CI Upper	% Difference
**Food Literacy Behaviours**						
**Plan & Manage** (n = 923)	8.96	9.82	<0.0001	−0.97	−0.77	9.68
**Selection** (n = 1050)	2.92	3.66	<0.0001	−0.81	−0.66	25.12
**Preparation** (n = 1022)	6.33	7.09	<0.0001	−0.64	−0.66	11.86
**Dietary Intake Behaviours**						
**Serves of fruit** (n = 1013)	1.58	1.822	<0.0001	−0.30	−0.18	15.06
**Serves of vegetables** (n = 1009)	2.32	2.88	<0.0001	−0.64	−0.49	24.33

CI: Confidence Interval.

**Table 3:** The number of participants reporting never, less than once a week, once or twice a week and three or more times a week relating to fast food meal frequency. Percentages refer to the percentage of the specific preprogram group.

Self-Reported Fast Food Meal Frequency (n = 1016)					
		Postprogram *			
		Never	Less than Once a Week	Once or Twice a Week	Three or More Times a Week
**Preprogram**	**Never**	(79.6%)	51 (16.2%)	10 (3.2%)	3 (1.0%)
	**Less than once a week**	116 (29.0%)	213 (53.3%)	66 (16.5%)	5 (1.3%)
	**Once or twice a week**	23 (9.3%)	87 (35.1%)	123 (49.6%)	15 (6.0%)
	**Three or more times a week**	4 (7.4%)	9 (16.7%)	25 (46.3%)	16 (29.6%)

* *p* < 0.0001.

**Table 4:** The number of participants reporting never, less than once a week, once or twice a week and three or more times a week relating to sugar-sweetened drink frequency. Percentages refer to the percentage of the specific preprogram group.

Self-Reported Sugar-Sweetened Drinks Frequency (n = 1017)					
		Postprogram *			
		Never	Less Than Once a Week	Once or Twice a Week	Three or More Times a Week
**Preprogram**	**Never**	440 (86.3%)	49 (9.6%)	17 (3.3%)	4 (0.8%)
	**Less than once a week**	84 (35.9%)	112 (47.9%)	33 (14.1%)	5 (2.1%)
	**Once or twice a week**	24 (16.8%)	35 (24.5%)	69 (48.3%)	15 (10.5%)
	**Three or more times a week**	14 (10.8%)	15 (11.5%)	36 (27.7%)	65 (50%)

* *p* < 0.0001.

**Table 5:** The number of participants in low, moderate and high scoring groups for the three food literacy behaviour factors (a) plan & manage, (b) selection and (c) preparation. Percentages refer to the percentage of the specific preprogram population (low, moderate or high).

**(a) Plan & Manage (n = 971)**				
		Postprogram *		
		**Low**	**Moderate**	**High**
**Preprogram**	**Low**	121 (39.0%)	110 (35.5%)	79 (25.5%)
	**Moderate**	22 (6.6%)	151 (45.2%)	161 (48.2%)
	**High**	4 (1.2%)	52 (15.9%)	271 (82.9%)
**(b) Selection (n = 1050)**				
		Postprogram *		
		**Low**	**Moderate**	**High**
**Preprogram**	**Low**	116 (26.1%)	158 (35.6%)	170 (38.3%)
	**Moderate**	28 (9.2%)	128 (42.1%)	148 (48.7%)
	**High**	17 (5.6%)	50 (16.6%)	235 (77.8%)
**(c) Preparation (n = 1022)**				
		Postprogram *		
		**Low**	**Moderate**	**High**
**Preprogram**	**Low**	121 (35.3%)	123 (35.9%)	99 (28.9%)
	**Moderate**	35 (9.3%)	165 (43.9%)	176 (46.8%)
	**High**	10 (3.3%)	56 (18.5%)	237 (78.2%)

* *p* < 0.0001.

**Table 6:** Demographic and dietary behaviours associated with food literacy behaviour improvement.

**Plan & Manage (n = 502)**	
**Change in self-reported sugar-sweetened drink intake**	
Increased intake	1
No change in intake	2.95 (1.30–6.66) *p* = 0.0094
Decreased intake	2.52 (1.00–6.346) *p* = 0.0499
**Change in self-reported vegetable intake** (serves/day)	1.37 (1.06–1.77) *p* = 0.0167
**Selection (n = 666)**	
**Sex**	
Female	1
Male	2.72 (1.10–6.76) *p* = 0.0308
**Household Composition**	
Single person	1
Couple with no children	4.72 (1.62–13.79) *p* = 0.0045
Single parent with children	1.06 (0.42–2.99) *p* = 0.8116
Couple with children	1.06 (0.47–2.39) *p* = 0.8836
Other (e.g., shared or supported accommodation, family or extended family)	1.93 (0.78–4.74) *p* = 0.1529
**Born in Australia ^1^**	
Yes	1
No	2.11 (1.14–3.92) *p* = 0.0176
**Reason for coming: Learn to read food labels**	
Not Selected	1
Selected	2.54 (1.39–4.65) *p* = 0.0025
**Reason for coming: Make healthier snacks and lunchboxes for children**	
Selected	1
Not selected	1.95 (1.07–3.55) *p* = 0.0290
**Preparation (n = 525)**	
**SEIFA ^2^**	
Low	1
Middle	0.73 (0.37–1.46) *p* = 0.3759
High	2.68 (1.24–5.78) *p* = 0.0118
**Responsibility for choosing and preparing the household meals**	
All	1
Some	1.53 (0.81–2.86) *p* = 0.1892
None	14.91 (1.57–141.69) *p* = 0.0187
**Change in self-reported sugar-sweetened drink intake**	
Increased intake	1
No change in intake	3.54 (1.49–8.41) *p* = 0.0041
Decreased intake	8.46 (2.96–24.17) *p* = 0.0001

^1^ Added in later version of questionnaire. ^2^ SEIFA, Socio-Economic Indexes for Areas, derived from postcode [34].
